# The 3D-Printed Honeycomb Metamaterials Tubes with Tunable Negative Poisson’s Ratio for High-Performance Static and Dynamic Mechanical Properties

**DOI:** 10.3390/ma14061353

**Published:** 2021-03-11

**Authors:** Chunxia Guo, Dong Zhao, Zhanli Liu, Qian Ding, Haoqiang Gao, Qun Yan, Yongtao Sun, Fuguang Ren

**Affiliations:** 1 Department of Mechanics, School of Science, Xi’an University of Architecture & Technology, Xi’an 710055, China; chunxiaguo@xauat.edu.cn (C.G.); zdxauat@163.com (D.Z.); 2 Applied Mechanics Laboratory, School of Aerospace Engineering, Tsinghua University, Beijing 100084, China; liuzhanli@Tsinghua.edu.cn; 3 Tianjin Key Laboratory of Nonlinear Dynamics and Control, Tianjin University, Tianjin 300350, China; qding@tju.edu.cn (Q.D.); hqgao@tju.edu.cn (H.G.); 4 Key Laboratory of Aeroacoustics and Dynamics, Aircraft Strength Research Institute, Xi’an 710065, China; qunyan_ac@163.com; 5 State Key Laboratory of Mechanical Behavior and System Safety of Traffic Engineering Structures, Shijiazhuang Tiedao University, Shijiazhuang 050043, China

**Keywords:** convex–concave honeycomb tube (CCHT), negative Poisson’s ratio (NPR), dynamic tests, energy absorption

## Abstract

The synthesized understanding of the mechanical properties of negative Poisson’s ratio (NPR) convex–concave honeycomb tubes (CCHTs) under quasi-static and dynamic compression loads is of great significance for their multifunctional applications in mechanical, aerospace, aircraft, and biomedical fields. In this paper, the quasi-static and dynamic compression tests of three kinds of 3D-printed NPR convex–concave honeycomb tubes are carried out. The sinusoidal honeycomb wall with equal mass is used to replace the cell wall structure of the conventional square honeycomb tube (CSHT). The influence of geometric morphology on the elastic modulus, peak force, energy absorption, and damage mode of the tube was discussed. The experimental results show that the NPR, peak force, failure mode, and energy absorption of CCHTs can be adjusted by changing the geometric topology of the sinusoidal element. Through the reasonable design of NPR, compared with the equal mass CSHTs, CCHTs could have the comprehensive advantages of relatively high stiffness and strength, enhanced energy absorption, and damage resistance. The results of this paper are expected to be meaningful for the optimization design of tubular structures widely used in mechanical, aerospace, vehicle, biomedical engineering, etc.

## 1. Introduction

With the development of science and technology, high-performance structural materials have become more and more desired in the engineering fields. In this regard, mechanical metamaterials are increasingly popular in the research community [1,2,3,4,5,6,7]. The negative Poisson’s ratio (NPR) tube is a typical mechanical metamaterial, which expands (shrinks) transversely under axial compression (tension). NPR tube has many advantages that the conventional tube with positive Poisson’s ratio (PPR) does not have, such as enhanced circumferential stiffness [8,9], the ability to undergo synclastic curvature [10,11], higher flexibility [12], increased resistance to kinking [13], etc.

The unique mechanical properties of the NPR tube make its application in mechanical, medical, aerospace, and other fields very attractive [14,15]. Regarding the applications of the NPR convex–concave honeycomb tubes (CCHTs), as in the vascular stent, for example [8,16,17,18], it is found that under axial compressive loadings, the stent will shrink inward, thus reducing the risk of vascular walls becoming overstretched and further injured. On the contrary, for the conventional vascular stent with a PPR, under axial compressive stresses, it will expand outward and thus exert extra forces to the vascular walls. This may bring more damage to the diseased region.

In order to explore its mechanical properties and promote its practical application in engineering, researchers have proposed various NPR tubes in recent decades. The fabrication of NPR tubes using soft lithography was first described by Bing Xu et al. [19]. The NPR tube used as angioplasty stents was first proposed by W.J.S. Dolla because it exhibits high circumferential strength in its expanded configuration and low flexural rigidity in its crimped configuration [8,9]. The characteristics of wave propagation, synclastic property, buckling behavior, and an analytical method for predicting the length–diameter relationship in re-entrant hexagonal NPR tubes are analyzed in literature [12,13,20,21]. Grima et al. systematically studied the mechanical properties of the NPR stent from rotating-square and found that the mechanical properties of the stent can be improved by designing the negative Poisson’s ratio [16,17,18,22,23]. Recently, more NPR stent structures have been proposed, such as folding tubular structures [24,25], V-type and X-type [26,27], tetrachiral and anti-tetrachiral hybrid [28], and chiral–anti-tetrachiral stents [29,30]. In addition to being used as a medical stent, the NPR tube can also be used as a jounce bumper in vehicle suspension due to its excellent energy absorption performances [31,32,33]. In recent years, there is more and more research on NPR tubes with their widespread use. The cylindrical auxetic double arrowed honeycomb was proposed, and its mechanical properties were studied theoretically [34] and experimentally [33,35]. The spatial tubular structure with NPR composed of square circular aperture arrays has been studied [36,37,38,39]. The impact performances under the axial force of the auxetic honeycomb cylindrical tube have been investigated [40,41,42]. Liu introduced deterministic routes to soft materials with isotropic tunable NPR over large strains [43]. A series of chiral-type auxetic cylindrical shells were proposed and the mechanical performances under axial compression have been evaluated [44,45,46,47,48,49]. However, there is no research on 3D CCHT as an energy absorber to enhance crash performance. Therefore, it is necessary to evaluate the 3D CCHTs in terms of the specific energy absorption under the axial crushing force from the perspective of energy absorption.

Compared with the various NPR tubes reported in the open literature, NPR CCHTs have the advantages of a simpler geometrical structure and the subsequent higher convenience of fabrication and application. However, the NPR effect of CCHTs is usually briefly mentioned in the reported literature, the detailed mechanical behavior of the NPR CCHTs under quasi-static and dynamic loading is still unclear. In fact, a systematic and deep understanding of quasi-static and dynamic loading behaviors of the NPR CCHTs is crucial to their practical applications. In addition, it is well known that geometric topology, i.e., the geometric parameters, could have a great influence on the mechanical behavior of metamaterials’ structures [50,51,52,53,54,55]. Nevertheless, reports on how will the geometrical parameters affect the mechanical properties of NPR CCHTs are still limited.

Taking into account the advantages of the NPR CCHTs mentioned above and the rapid development of the 3D-printed technology, this paper aims to explore systematically the mechanical behaviors of NPR CCHTs under quasi-static and dynamic loading conditions through experimental methods. Based on the nylon materials, one 3D-printed conventional square honeycomb tube (CSHT) and three kinds of CCHTs with equal mass have been chosen as examples for illustration. Two axial compressive loading speeds, 1 mm/min and 5 m/s, are selected to demonstrate mechanical behaviors of the PPR CSHT and NPR CCHT under static and dynamic loading conditions, respectively. In this paper, the mechanical properties of CCHTs are studied, including elastic modulus, peak force, energy absorption, and damage mode.

The research in this study is expected to provide a theoretical basis for the optimal design of CCHT, so as to promote this engineering application in the fields of mechanical, medical, aviation, and aerospace industries.

## 2. Samples, Quasi-Static, and Dynamic Loading Tests of the 3D-Printed CCHT

### 2.1. Geometric Topology and 3D-Printed Samples of the CCHT

**Figure 1:**
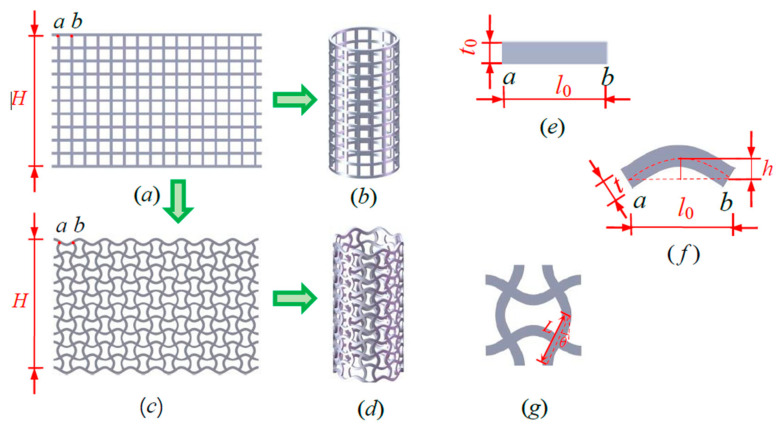
Schematic of (**a**) the square honeycomb lattices, (**b**) the square honeycomb tubes (CSHTs), (**c**) the convex–concave lattices, (**d**) the negative Poisson’s ratio (NPR) convex–concave honeycomb tubes (CCHTs), (**e**) the straight cell walls of (**a**), (**f**) the sinusoidal beams of (**c**), and (**g**) the unit cell of (**d**).

The conventional square honeycomb tubes (CSHTs), shown in Figure 1b, are constructed by rolling the square honeycomb lattices (Figure 1a). NPR CCHTs (Figure 1d) are constructed by rolling the convex–concave honeycomb lattices (Figure 1c), which are generated by replacing the straight cell walls (Figure 1e) of the square honeycomb lattices (Figure 1a) with the equal-mass curved sinusoidal beams (Figure 1f).

*t*_0_ and *l*_0_ (Figure 1e) are defined as the cell wall thickness and length of the square honeycomb lattices (Figure 1a). *B* is the out-of-plane thickness of the square honeycomb lattices. *W* and *H* are the width and height of the square honeycomb lattices. *t* and *h* (Figure 1f) are the cell wall thickness and chord height of the curved sinusoidal beams. The shape of the curved sinusoidal beam shown in Figure 1f could be mathematically expressed by

(1) $$z=h\sin\left(\frac{\pi }{l_{0}}y\right)y\in [0,\;l_{0}]$$

Defining the curve length of the curved sinusoidal beam as *s*, *s* is expressed as

(2) $$s=\int_{0}^{l_{0}}\sqrt{1+{\left(z^{\prime }\right)}^{2}}dx=\int_{0}^{l_{0}}\sqrt{1+h^{2}\pi^{2}/l_{0}^{2}\cos^{2}\left(\pi /l_{0}x\right)}dx.$$

According to the equal-mass principle, we have *l*_0_*t*_0_*B* = *stB*, which yields the cell wall thickness *t* of the curved sinusoidal beams as follows:

(3) $$t=t_{0}l_{0}/\int_{0}^{l_{0}}\sqrt{1+h^{2}\pi^{2}/l_{0}^{2}\cos^{2}\left(\pi /l_{0}x\right)}dx.$$

**Figure 2:**
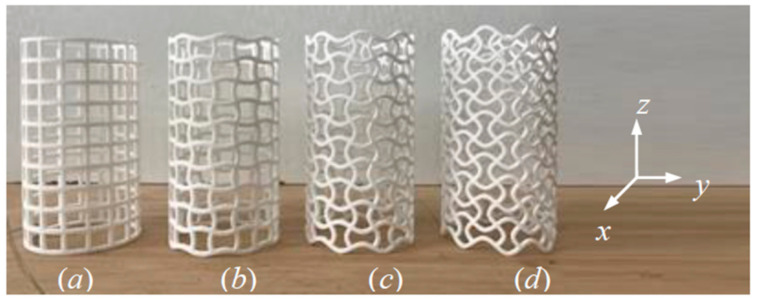
The 3D-printed experimental samples using the nylon materials: (**a**) CSHT0, (**b**) CCHT1, (**c**) CCHT2, and (**d**) CCHT3.

To show the static and dynamic loading behaviors of CCHTs, four kinds of samples, including one CSHT (Figure 2a), named CSHT0, and three kinds of equal-mass CCHTs corresponding to the CSHT, named as CCHT1, CCHT2, and CCHT3 (Figure 2b–d), are chosen as the examples for illustration. The four kinds of samples are tested under axial quasi-static (1 mm/min) and dynamic (5 m/s) compressive experiments. They are 3D-printed using nylon material with a density of 880 kg/m^3^ and an elastic modulus of 1.25 GPa. The samples were additively manufactured using an EOS P 500 system (Frankfurt, Germany), which uses a laser as the power source to sinter powdered nylon to build up the tubes. The laser selectively fuses powdered nylon by scanning cross sections generated from a 3D digital model, which is modeled by SolidWorks (2019). After each cross section is scanned, the powder bed is lowered by one layer thickness, a new layer of material is applied on top, and the process is repeated until the tube is completed. The masses of the four kinds of samples are all 10.5 g.

The parameters for CSHT0 are *t*_0_ = 2 mm, *l*_0_ = 10 mm, *B* = 2 mm, *W* = 160 mm, and *H* = 100 mm. The parameters for CCHT1, CCHT2, CCHT3 are *l*_0_ = 10 mm, *B* = 2 mm, H = 100 mm, and *h* = 1, 2, and 3 mm, respectively. Evidently, substituting *t*_0_ = 2 mm, *l*_0_ = 10 mm, *h* = 1, 2, and 3 mm into Equation (3) yields the curved cell wall thicknesses *t* of CCHT1, CCHT2, and CCHT3. They are 1.95 mm, 1.83 mm, and 1.67 mm, respectively. Here, the parameter *h/l*_0_, i.e., the ratio of the curved sinusoidal beams (Figure 1f) in convex–concave honeycomb lattices (Figure 1c), is utilized for describing the curved degree of the curved cell wall of CCHT. Evidently, the parameters *h/l*_0_ corresponding to CSHT, CCHT1, CCHT2 and CCHT3 are 0, 0.1, 0.2 and 0.3, respectively. These geometric parameters are shown in Table 1.

### 2.2. Quasi-Static and Dynamic Compressive Experiments Devices

**Figure 3:**
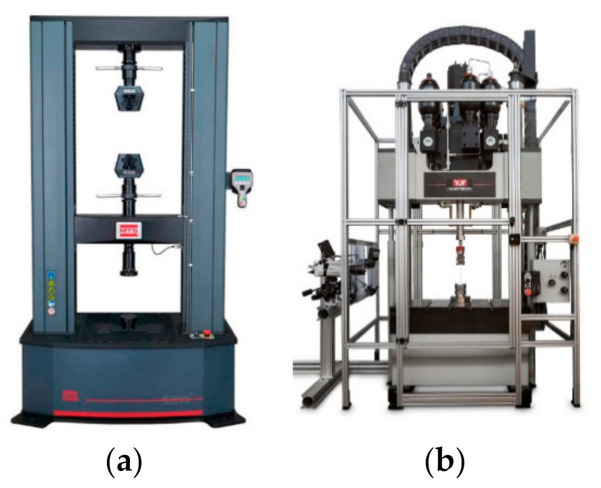
The quasi-static and dynamic compressive test machine: (**a**) MTS 810 testing machine; (**b**) Instron VHS high rate testing system machine.

Quasi-static compressive tests of CSHT0, CCHT1, CCHT2, and CCHT3 are conducted on the MTS 810 testing machine (MTS Systems Corporation, Eden Prairie, MN, USA) at Tianjin University (Figure 3a). Specimens are put on the lower platen. In the loading process, the lower platen moves upward to crush the specimens. The compressive loading velocity is 1 mm/min (1.67 × 10^−5^ m/s). For each sample, the quasi-static compressive tests are repeated three times.

Dynamic compressive tests of CSHT0, CCHT1, CCHT2, and CCHT3 are conducted on the Instron VHS (Video Home System) high-rate testing system (Instron Corporation, Norwood, MA, USA) at Tianjin University (Figure 3b). The specimens are placed on a fixed lower plate. The upper plate moves down to impact the specimens. Some glue is applied between the specimens and the lower fixed platen to avoid the slip of the specimens during the compressive crushing process. The compressive loading velocity is 5 m/s. For each sample, the dynamic compressive tests are also repeated three times.

### 2.3. Repeatability of the Experimental Force–Displacement Curve

**Figure 4:**
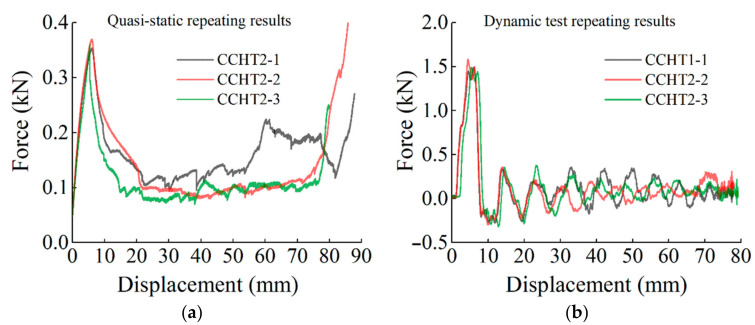
Three-times repeated force–displacement curves of the samples under quasi-static and dynamic compressive loading conditions: (**a**) CCHT2 under a quasi-static loading speed of 1 mm/min and (**b**) CCHT1 under a dynamic loading speed of 5 m/s^−1^.

In this part, the repeatability of the static and dynamic compressive experiments is shown. The three times repeated force–displacement curves are presented in Figure 4. From Figure 4, we can derive that although the measured data of peak forces have slight dispersity, the forces in the plateau region are of good repeatability in general, which indicates that experimental results of the quasi-static and dynamic compression are reliable.

## 3. Mechanical Properties of the NPR CCHT under Static Loading Conditions

This section investigates the mechanical properties of the NPR CCHT under static loading conditions and systematically studies the effects of geometrical morphology on elastic modulus, damage modes, and energy absorption properties.

### 3.1. Effect of Geometrical Morphology on Elastic Moduli of the CCHT

The effects of the CSHT0 (*h*/*l*_0_ = 0), CCHT1 (*h*/*l*_0_ = 0.1), CCHT2 (*h*/*l*_0_ = 0.2) and CCHT3 (*h*/*l*_0_ = 0.3) are investigated in this part.

The auxetic structure micromechanically behaves as a planar orthotropic lamina even though its microscopic mechanical behavior is highly anisotropic [9]. Axial stiffnesses $E_{z}$ of PPR CSHT and NPR CCHT can be approximated as follows [56,57]:

(4) $$E_{z}=\left\{\begin{array}{ll} \frac{t_{0}}{l_{0}}E_{s} & \mathrm{for}\;\mathrm{the}\;\mathrm{PPR}\;\mathrm{CSHT}\\ \frac{\frac{2}{L\cos\theta }}{\frac{5\sin^{2}\theta }{8K_{f}}+\frac{\sin^{2}\theta }{K_{h}}+\frac{\cos^{2}\theta }{K_{s}}} & \mathrm{for}\;\mathrm{the}\;\mathrm{NPR}\;\mathrm{CCHT} \end{array}\right.$$

where $K_{f}=\frac{E_{s}Bt^{3}}{L^{3}}\;\;K_{h}=\frac{\gamma G_{s}Bt}{L}\;\;\;K_{s}=\frac{E_{s}Bt}{L}\;$

*E_S_* and *G_S_* are Young’s modulus and shear modulus of the solid of which the CCHT is made (*E_s_ =* 1250 MPa). For the square cross section of struts, the geometrical factor is *γ =* 5/6. The parameter *L* is 10.3 mm, 10.9 mm, 11.9 mm, respectively, for CCHT1, CCHT2, and CCHT3. The parameter *θ* for CCHT1, CCHT2 and CCHT3 is 11.3°, 21.9° and 31°.

The Poisson’s ratio could be calculated as [56,57]

(5) $$\nu_{xz}=\nu_{zx}=\left\{\begin{array}{ll} \frac{t_{0}}{l_{0}}\nu_{s} & \mathrm{for}\;\mathrm{the}\;\mathrm{PPR}\;\mathrm{CSHT}\\ -\frac{\frac{3\sin^{2}\theta_{i}}{8K_{f}}}{\frac{5\sin^{2}\theta_{i}}{8K_{f}}+\frac{\sin^{2}\theta_{i}}{K_{h}}+\frac{\cos^{2}\theta_{i}}{K_{s}}} & \mathrm{for}\;\mathrm{the}\;\mathrm{NPR}\;\mathrm{CCHT} \end{array}\right.$$

where *ν*_s_ is the Poisson’s ratio of the solid of which the CCHT is made (*ν*_S_ = 0.28).

**Figure 5:**
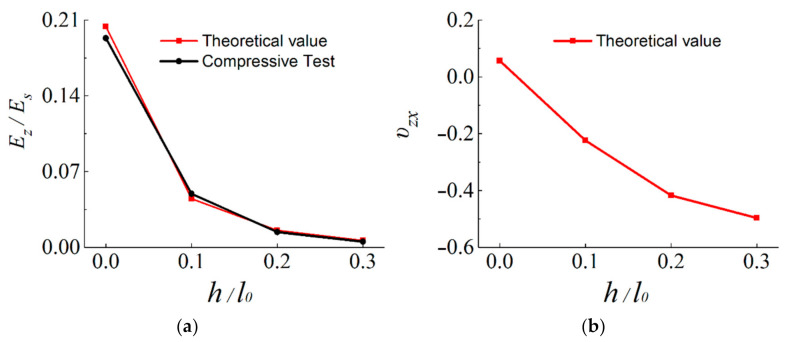
(**a**) Relative Young’s modulus *E_z_/E_s_* of the CSHT0, CCHT1, CCHT2, and CCHT3 and (**b**) Poisson’s ratio *ν*_zx_ (*ν*_zx_ = *ν*_xz_).

The relative Young’s modulus $E_{z}/E_{s}$ and Poisson’s ratio data obtained from Equations (4) and (5) are plotted as shown in Figure 5.

Figure 5a shows the ratio *E_z_/E_s_* of the four samples studied by theoretical and experimental methods. *E_z_* values of the four samples by the theoretical method are calculated through Equation (4). From Figure 5a, it can be observed that on the whole, the ratio *E_z_*/*E_s_* calculated by the experimental method is in good agreement with the theoretical results. Moreover, the relative Young’s modulus *E_z_/E_s_* increase with the decreases of the ratio *h/l*_0_ of the unit cell.

It can be clearly derived from Figure 5b that *ν*_zx_ of CSHT0, CCHT1, CCHT2, and CCHT3 are 0.057, −0.22, −0.42, and −0.5, respectively. Obviously, CSHT0 with straight cell edges has PPR, while the CCHT with sinusoidal cell edges has NPR, and with the increase of the CCHT ratio *h/l*_0_, the NPR effect accelerates. That is to say, as the cell edge changes from straight (CSHT0) to curved sinusoidal (CCHT), Poisson’s ratios change from positive to negative. Moreover, the larger the CCHT cell’s curvature is, the more obvious its NPR effect. This indicates that the CCHT’s NPR can be adjusted by changing the ratio *h/l*_0_.

### 3.2. Damage Patterns and Energy Absorption Properties

For quasit-static compression, all specimens experienced a rapidly increasing elastic zone, a relatively smooth platform collapse zone, and a rapidly increasing densification zone of force with displacement. During the elastic region, the load value increases rapidly to an initial peak, indicating the localization of the first plastic folding. Then, continuous plastic folding mechanisms characterize the average crushing force (platform). The energy absorption performance is expressed as

(6) $$W=\int_{0}^{L}F(x)dx$$

where *L* is the effective total length, and *F* is the crushing force.

**Figure 6:**
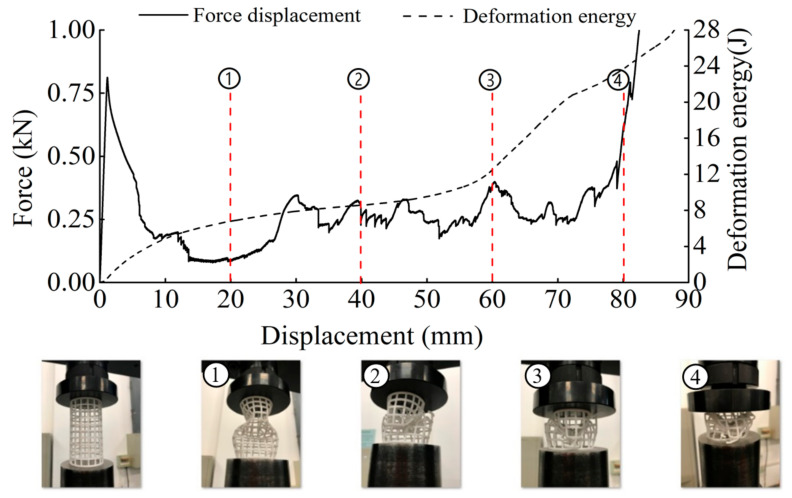
Deformation process of CSHT0 (1–4 stages of deformation during quasi-static compression test).

**Figure 7:**
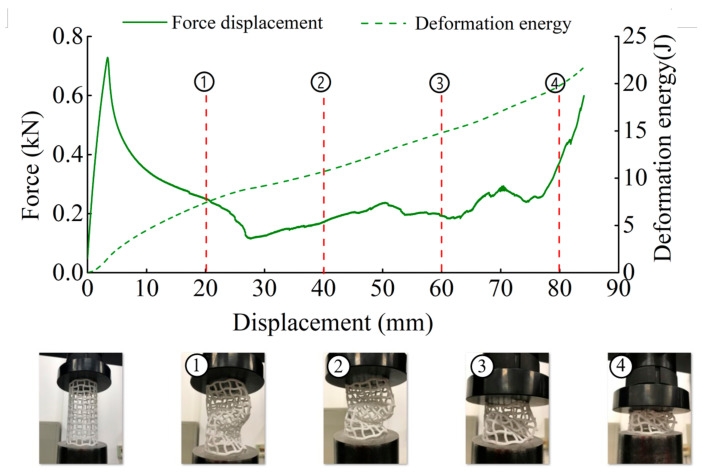
Deformation process of CCHT1 (1–4 stages of deformation during quasi-static compression test).

**Figure 8:**
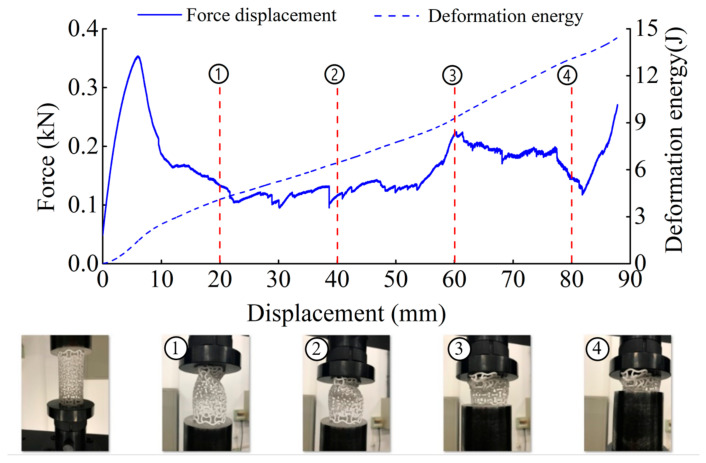
Deformation process of CCHT2 (1–4 stages of deformation during quasi-static compression test).

**Figure 9:**
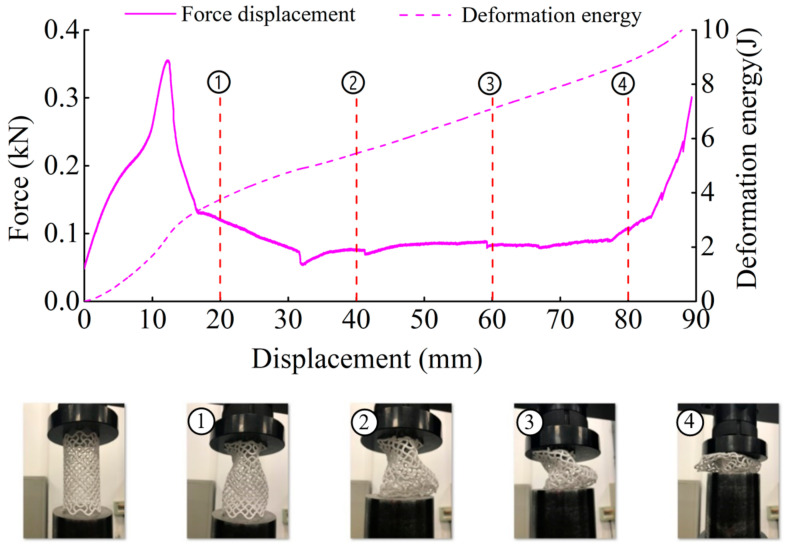
Deformation process of CCHT3 (1–4 stages of deformation during quasi-static compression test).

The deformation in the elastic region is uniform and not uniform in the plateau collapse zone. The progressive buckling starts from the middle region of the specimen. Figure 6, Figure 7, Figure 8 and Figure 9 display the detailed deformation process of the tubes under quasi-static compression.

**Figure 10:**
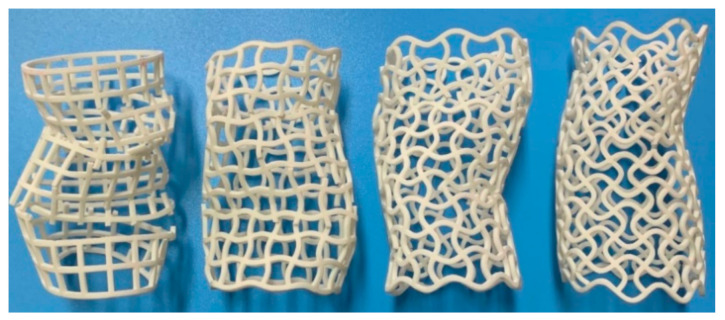
The tubes after unloading.

From Figure 6, Figure 7, Figure 8 and Figure 9, it is observed that there is a long collapse platform in the loading process for all the tubes due to the cellular structure properties. However, depending on the ratio *h/l*_0_, the damage pattern is quite different. Because of the good elasticity of nylon material, the elastic deformation recovered after unloading. The shapes of the four types of tubes after unloading are shown in Figure 10. It could be observed from Figure 10 that CSHT0 with the PPR is the most destructive, while the damage degree of the NPR CCHT is lighter and decreases with the increase of the ratio *h/l*_0_. In other words, the NPR CCHT is softer and has more deformation ability in contrast with the CSHT0 of the same mass.

**Figure 11:**
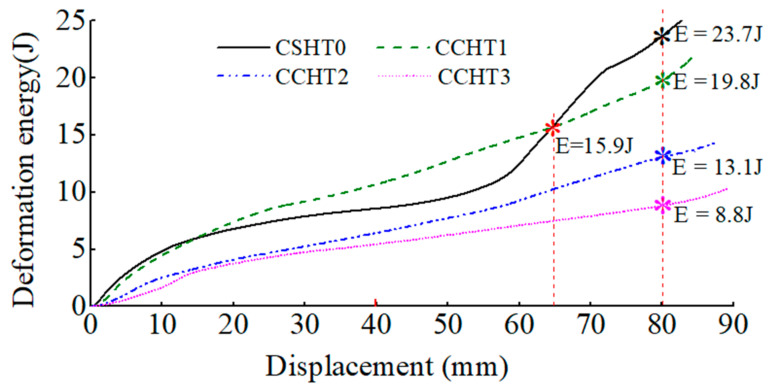
Comparison of deformation energy curves of the CCHT0, CCHT1, CCHT2, and CCHT3 under quasi-static loading conditions.

The deformation energy curves of four kinds of tubes are plotted in Figure 11. The energy increases with the increase of displacement. When quasi-static compression produces the same displacement, the energy absorbed by the four types of samples is different. For the cases in which the displacement is 5 mm, 15 mm, 40 mm, and 65 mm, respectively, the energy absorbed by the four types of samples is shown in Table 2.

**Figure 12:**
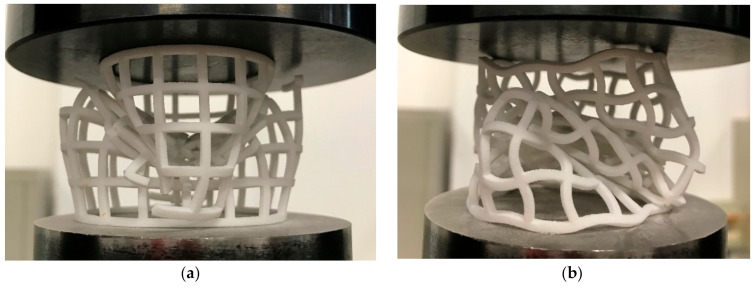
(**a**) CCHT0 at the displacement of 65 mm under quasi-static test. (**b**) CCHT1 at the displacement of 65 mm under quasi-static test.

Figure 11 shows that the absorption energy of NPR CCHT1 is larger than that of the PPR CSHT0 before the displacement of about 65 mm. The CSHT0 absorbed more energy than CCHT1 after displacement 65mm because it broke and stacked together as a solid tube. Although CCHT1 is also compacted, due to its curved contact with the indenter, when its displacement exceeds 65mm, the contact between the tube and the indenter deviates somewhat, resulting in buckling of both the whole tube and the local tube, which may be the reason for the decrease of its energy absorption effect. (see Figure 12). With the increase of the NPR, the tube is more prone to deformation. However, it can be seen from Table 2 that with the increase of h/*l*_0_ of the CCHT, the energy absorption gradually decreases.

From the above analysis, it can be concluded that the energy absorption capacity of the CCHT can be adjusted and enhanced by reasonably designing the ratio *h/l*_0_, i.e., the geometrical shape of the CCHT. That is to say, by slightly introducing the NPR effect, the CCHT’s energy absorption capacity is enhanced with only slightly reducing the stiffness and strength.

## 4. Mechanical Properties of the NPR CCHT under Dynamic Loading Conditions

In this part, dynamic properties of the NPR CCHTs under dynamic loading conditions are systematically analyzed in terms of dynamic enhancement, damage patterns, and energy absorption properties.

### 4.1. Dynamic Enhancement Analysis

**Figure 13:**
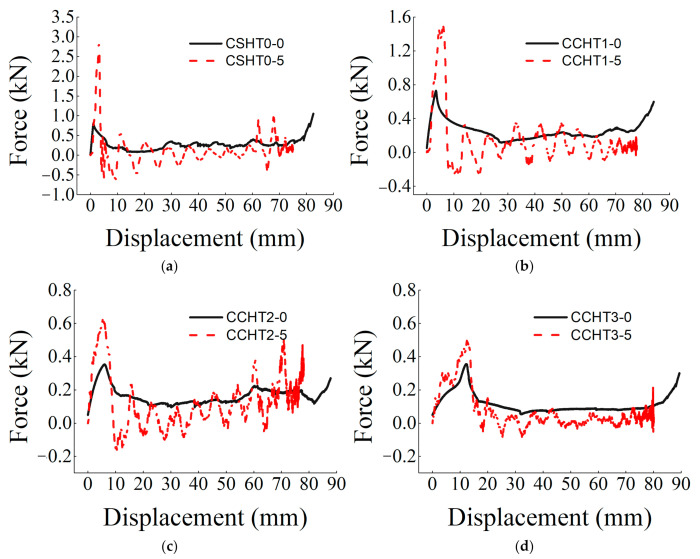
Comparison of the force–displacement curves under quasi static and dynamic conditions: (**a**) CSHT0, (**b**) CCHT1, (**c**) CCHT2, and (**d**) CCHT3.

The force–displacement curves of CSHT0, CCHT1, CCHT2, and CCHT3 under dynamic compression tests are shown in Figure 13. To analyze the dynamic enhancement, the responses under quasi-static compression loading are also plotted in Figure 13.

For all the dynamic tests, it could be observed from Figure 13 that when the force exceeds the peak, the force of all curves drops to a smaller value and then begins to fluctuate with the displacement increasing. Unlike quasi-static tests, no plateau collapse region could be detected. One possible reason is that the nylon tubes fracture layer by layer under impact compression and do not exhibit yielding characteristics. Section 4.2 describes the deformation process in detail.

**Figure 14:**
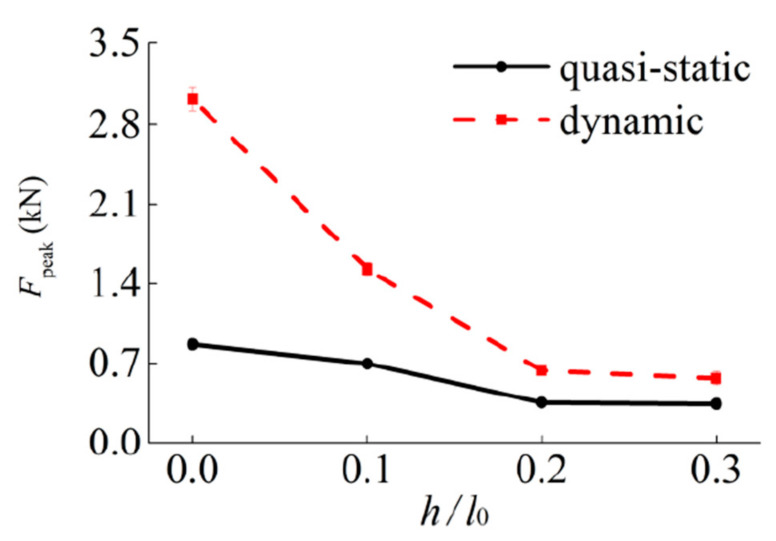
The initial peak force under quasi-static and dynamic compressive loading.

The initial peak force of the tube is used to evaluate the strengthening effect of the NPR tubes. The four kinds of samples’ initial peak force under quasi-static and dynamic compression loading are shown in Figure 14. It can be seen that the strength of the four samples all increases with the decrease of the ratio *h/l*_0_ under compressive loading.

To study and quantify the dynamic enhancement, introduce a dynamic enhancement rate *γ* [58]

(7) $$\gamma =\frac{F_{Dyn}-F_{Qs}}{F_{Qs}},$$

where *F_Dyn_* and *F_Qs_* are the initial peak force in the dynamic case and quasi-static case, respectively.

Based on the experimental data, the dynamic enhancement rate *γ* is listed in Table 3.

As shown in Table 1 and Figure 14, the velocity has a great influence on the out-of-plane peak force. The dynamic enhancement rate is 276%, 118%, 76.2% and 63.6% for tubes CSHT0, CCHT1, CCHT2 and CCHT3, respectively. The increase of conventional tubes CSHT0 is the largest, up to 276%. This shows that the conventional tubes are more sensitive to velocity. It is worth noting that CCHT2 and CCHT3 have almost the same peak force in the quasi-static test. However, with the increase of velocity, the difference between them is increasing.

### 4.2. Damage Patterns and Energy Absorption Properties

**Figure 15:**
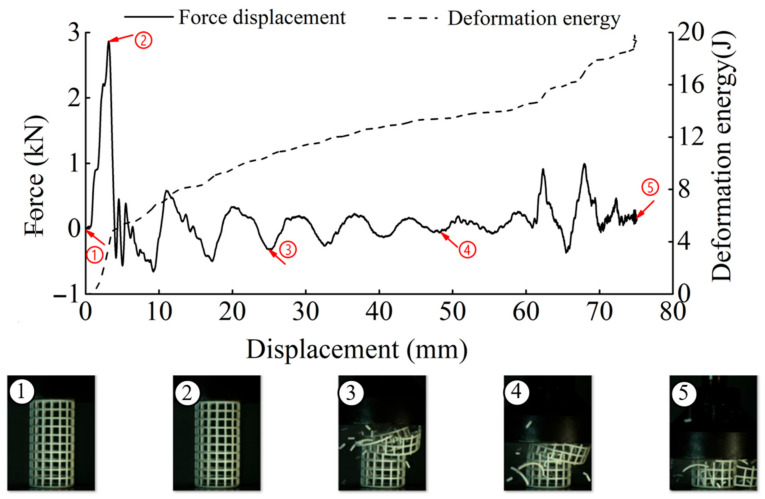
The results of dynamic tests of CSHT0 (1–5 stages of deformation during the dynamic compressive test).

**Figure 16:**
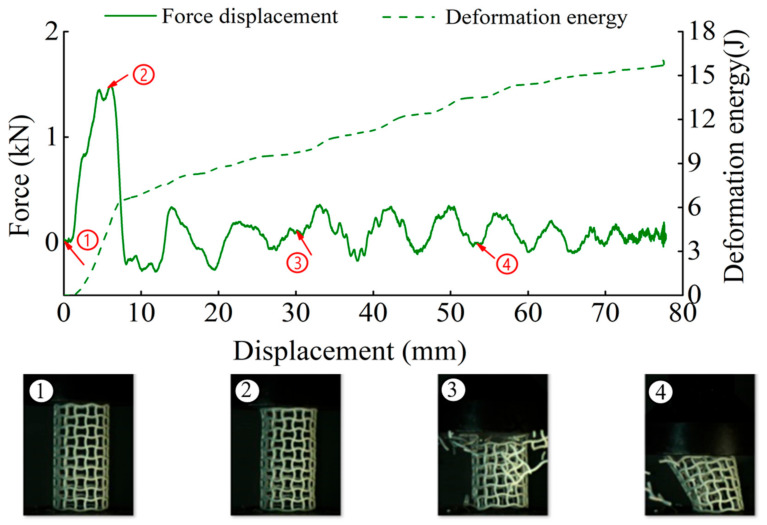
The results of dynamic tests of CCHT1 (1–4 stages of deformation during the dynamic compressive test).

**Figure 17:**
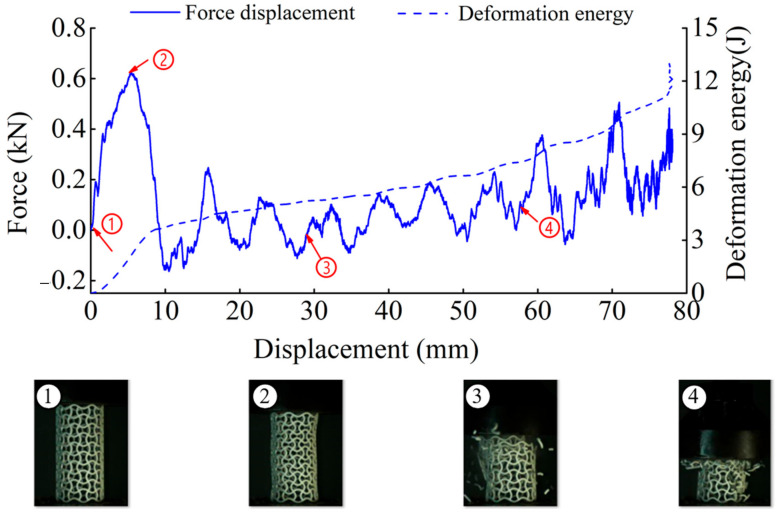
The results of dynamic tests of CCHT2 (1–4 stages of deformation during the dynamic compressive test).

**Figure 18:**
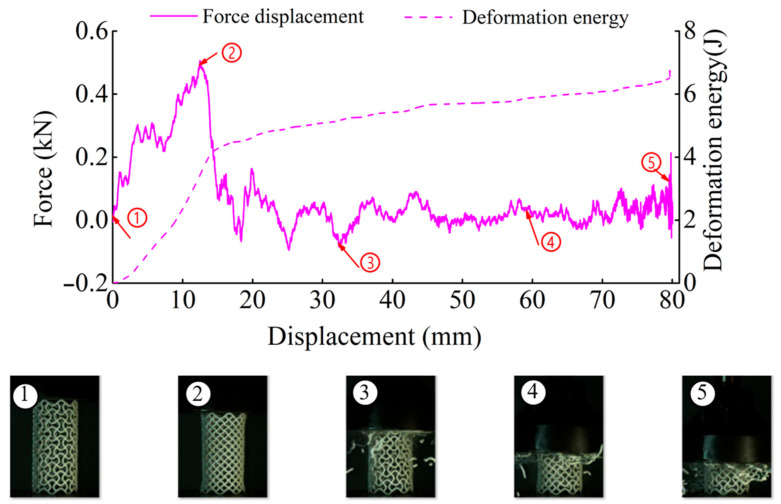
The results of dynamic tests of CCHT3 (1–5 stages of deformation during the dynamic compressive test).

In order to understand the damage patterns of tubes under dynamic loading, a digital camera (Instron Corporation, Norwood, MA, USA) and a high-speed camera (Instron Corporation, Norwood, MA, USA) were used. Figure 15, Figure 16, Figure 17 and Figure 18 display the detailed deformation process of the tubes under dynamic loading.

For dynamic compression, the failure mode of these tests exhibits brittle crushing. In compression, the cells suffer progressive crushing. Figure 15 shows the deformation process of tube CSHT0 under velocity 5 m/s. It can be observed from Figure 15 that the loading deformation first occurs at the proximal end and then extends downward. The deformation process involves a sequence of fracture events. First, the weakest rod bends and then breaks. The broken fragment falls off. The specimen is broken layer by layer until the impact ends. The magnitude of local deformation in the top contact region is greater than that in the bottom region, which shows that the inertia force in the accelerating process causes obvious deformation of the specimen.

Figure 16, Figure 17 and Figure 18 show the deformation process of CCHT1, CCHT2, and CCHT3 under a compressive velocity of 5 m/s. It can be observed that the deformation of NPR tubes is slightly different from the conventional tube. From Figure 18 we can clearly derive that the elastic deformation occurred first, then the first layer contact with the impact platen broke, the lower part rebounded, and the contact area continued to fracture, the lower part restored, then repeated the deformation process elastic deformation–fracture–rebound–fracture–restore, until the last layer was broken. In Figure 15 and Figure 16, the deformation process can also be observed, even though less obvious than in Figure 18.

In Figure 15, Figure 16, Figure 17 and Figure 18, we can detect a negative value of the force after the peak force. This may be due to the first row of cracks in the structure as a result of the tensile damage mechanism caused by the rebound effect of the upper plate. The main reason for this situation is the local defects such as micro cracks or pores in the samples.

**Figure 19:**
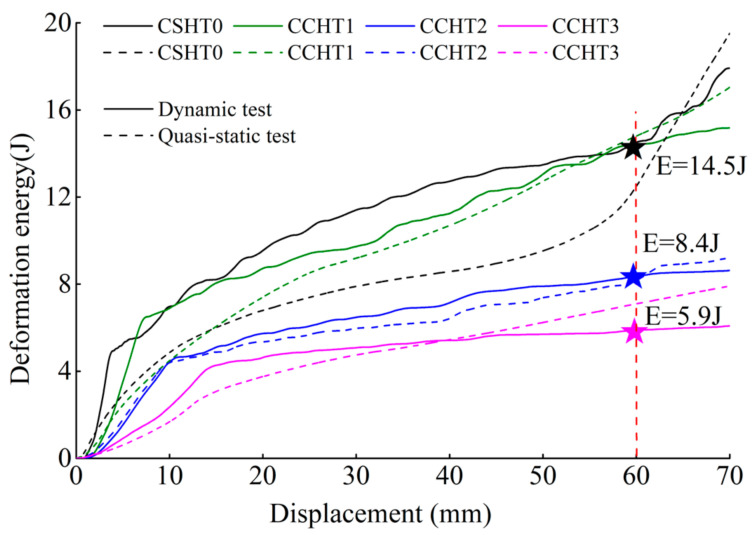
The comparison of deformation energy between dynamic and quasi-static compressive tests for CSHT0, CCHT1, CCHT2, and CCHT3.

The deformation energy curves of four kinds of tubes under quasi-static and dynamic tests are plotted in Figure 19. It can be observed that the deformation energy value under the dynamic test is obviously higher than the quasi-static test results. The CSHT0 specimens show a high deformation rate sensitivity, while the NPR CCHT2 and CCHT3 specimens are almost insensitive to deformation rate effects.

For the cases in which the displacement is 5 mm, 15 mm, 40 mm, and 60 mm, respectively, the energy absorbed by the four types of samples under dynamic test is shown in Table 4.

**Figure 20:**
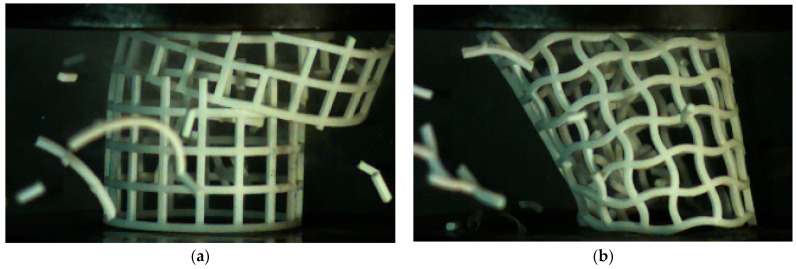
(**a**) CCHT0 at the displacement of 60 mm under dynamic test. (**b**) CCHT1 at the displacement of 60 mm under dynamic test.

From Figure 19 and Table 4, we can derive that CCHT1 did not show better energy absorption than CSHT0 as in the quasi-static test. This may be because CCHT1 is more prone to tilt during the impact process due to its sinusoidal contact surface (Figure 20). This effect can be eliminated by adding a plane at the upper and lower edges of the tube, which will be analyzed further in the follow-up study.

## 5. Conclusions

NPR tube is a typical metamaterial, which has great potential in the mechanical, vehicle, and biomedical engineering. A comprehensive understanding of its mechanical properties plays a very important role in its diversified applications. In this paper, the quasi-static and dynamic crushing behaviors focusing on the initial peak force and energy absorption of four kinds of open-cell tubes with equal mass and different *h/l*_0_ ratios are investigated experimentally based on the polymer materials nylon. The dynamic enhancement rate and energy absorption are depending on the ratio *h/l*_0_. The ratio *h/l*_0_ ratio is a key parameter for designing the NPR tube to express its inherent properties. The NPR could be rational design by adjusting the *h/l*_0_ ratio and could have relatively high stiffness, strength, and damage resistance. In medicine, a small NPR tube is expected to be used for angioplasty stents, which contract radially as well as axially, thus passing through the arteries smoothly and reducing the risk of surgery. In the construction industry, it can be imagined that the NPR tube can be used as the expansion pipe of the expansion screw. As long as the expansion pipe is designed as an NPR pipe, it can make use of its own mechanical properties to promote the expansion of the pipe. The research of this paper fills a gap in existing research, provides the foundations for the practical applications of NPR tubes in the engineering fields. However, for the sake of gaining a systematic understanding, a further FE numerical study on mechanical properties of the NPR CCHT with different topologies and materials is required.

Noteworthy is that the study only focuses on static and dynamic loading behaviors of the 3D-printed nylon NPR CCHT. Static and dynamic loading behaviors of metal NPR CCHTs with higher stiffness and strength will be studied in future research.

## Figures and Tables

**Table 1:** The geometric parameters of the specimens.

Specimen	*t* (mm)	*h* (mm)	*l*_0_ (mm)	*B* (mm)	*h/l*_0_
CSHT0	2	0	10	2	0
CCHT1	1.95	1	10	2	0.1
CCHT2	1.83	2	10	2	0.2
CCHT3	1.67	3	10	2	0.3

**Table 2:** The energy absorbed by the specimen when the displacement is 5 mm, 15 mm, 40 mm, and 65 mm.

Specimen	*h/l*_0_	*W* (5 mm)	*W* (15 mm)	*W* (40 mm)	*W* (65 mm)
CSHT0	0	2.9J	6.1 J	8.5 J	15.9 J
CCHT1	0.1	2.4 J	6.1 J	10.7 J	15.9 J
CCHT2	0.2	1.1 J	3.4 J	6.5 J	10.2 J
CCHT3	0.3	0.6 J	2.9 J	5.4 J	7.6 J

**Table 3:** Comparison between quasi-static and dynamic initial peak force.

Specimen	*F*_Qs_ (kN)	*F*_Dyn_ (kN)	*γ*
CSHT0	0.864	3.248	276%
CCHT1	0.698	1.524	118%
CCHT2	0.362	0.638	76.2%
CCHT3	0.349	0.571	63.6%

**Table 4:** The energy absorbed by the specimen when the displacement is 5 mm, 15 mm, 40 mm, and 60 mm under dynamic test.

Specimen	*h/l*_0_	*W* (5 mm)	*W* (15 mm)	*W* (40 mm)	*W* (60 mm)
CSHT0	0	5.2 J	8.2 J	12.7 J	14.5 J
CCHT1	0.1	3.3 J	8.0 J	11.2 J	14.5 J
CCHT2	0.2	1.4 J	5.2 J	7.2 J	8.4 J
CCHT3	0.3	0.9 J	4.2 J	5.4 J	5.9 J

## Data Availability

Data is contained within the article.
